# Are Management Strategies Associated with Tolerance Acquisition in Infants with Cow’s Milk-Induced Allergic Proctocolitis?

**DOI:** 10.3390/jcm15103862

**Published:** 2026-05-17

**Authors:** Asena Pinar Sefer, Melek Yorgun Altunbas, Mehmet Sirin Kaya, Sumeyye Baysal, Hakan Kot, Ayse Senay Sasihuseyinoglu, Yasin Karali, Ezgi Yalcin Gungoren, Sevtap Barca, Yavuz Selim Ayhan, Elif Karakoc-Aydiner

**Affiliations:** 1 Division of Allergy and Immunology, Department of Pediatrics, School of Medicine, Recep Tayyip Erdogan University, Rize 53100, Türkiye; 2 Department of Pediatric Allergy and Immunology, Faculty of Medicine, Marmara University, Istanbul 34722, Türkiye; 3 Istanbul Jeffrey Modell Diagnostic Center for Primary Immunodeficiency Diseases, Istanbul 34899, Türkiye; 4 Isil Berat Barlan Center for Translational Medicine, Istanbul 34899, Türkiye; 5 Immune Deficiency Research and Application Center, Marmara University Hospital Center of Excellence, European Academy of Allergy and Clinical Immunology, Istanbul 34899, Türkiye; 6 Division of Allergy and Immunology, Department of Pediatrics, Sanliurfa Research and Training Hospital, Sanliurfa 63300, Türkiye; 7 Division of Allergy and Immunology, Department of Pediatrics, Balikesir Ataturk City Hospital, Balikesir 10100, Türkiye; 8 Division of Allergy and Immunology, Department of Pediatrics, School of Medicine, Atlas University, Istanbul 34408, Türkiye; 9 Division of Allergy and Immunology, Department of Pediatrics, Faculty of Medicine, Bursa Uludag University, Bursa 16059, Türkiye; 10 Division of Allergy and Immunology, Department of Pediatrics, Sisli Hamidiye Etfal Training and Research Hospital, University of Health Sciences, Istanbul 34668, Türkiye; 11 Department of Pediatrics, School of Medicine, Recep Tayyip Erdogan University, Rize 53100, Türkiye; 12 Department of Pediatrics, Sanliurfa Research and Training Hospital, Sanliurfa 63330, Türkiye

**Keywords:** breastfeeding, cow’s milk, elimination, food protein-induced allergic proctocolitis, infant, non-IgE-mediated food allergy, oral tolerance

## Abstract

**Background:** Food protein-induced allergic proctocolitis (FPIAP) is generally considered a benign and self-limited condition; however, both its natural course and the impact of management strategies on prognosis remain controversial. Data on modifiable factors influencing tolerance acquisition are limited. **Methods:** We conducted a retrospective cohort study including 180 infants with cow’s milk-induced FPIAP. Clinical characteristics, management strategies, and outcomes were analysed. Logistic regression was used to identify factors associated with delayed tolerance, and Kaplan–Meier and Cox regression analyses were performed to evaluate time to tolerance. **Results:** Tolerance was achieved in 91.2% of infants, with a median time from diagnosis to tolerance of 31.1 weeks. In multivariable logistic regression, multi-food elimination at presentation (OR, 2.58; 95% CI, 1.02–6.54; *p* = 0.046) and a longer interval from diagnosis to reintroduction (OR per week, 1.08; 95% CI, 1.02–1.14; *p* = 0.022) were independently associated with delayed tolerance. Exclusive breastfeeding was associated with lower odds of delayed tolerance in univariable analysis but not after adjustment. In unadjusted time-to-event analyses, observation-first management was associated with earlier tolerance acquisition (HR, 0.37; 95% CI, 0.22–0.62; *p* < 0.001), whereas multiple food allergy was associated with a lower probability of tolerance acquisition over time (HR, 0.60; 95% CI, 0.41–0.88; *p* = 0.009). Feeding modality also showed an unadjusted temporal association with tolerance acquisition, with exclusively breastfed infants demonstrating a more favorable pattern than formula-fed infants. **Conclusions:** The course of FPIAP appears to be influenced not only by clinical characteristics but also by management strategies. Delayed reintroduction and multi-food elimination were associated with later tolerance, while observation-first management was associated with earlier tolerance acquisition. These findings suggest that commonly used strategies such as prolonged elimination or delayed reintroduction may warrant reconsideration in selected infants and support a more individualized and less restrictive approach to management.

## 1. Introduction

Food protein-induced allergic proctocolitis (FPIAP) is one of the most common non-IgE-mediated gastrointestinal food allergies in early infancy. It typically presents in the first months of life with rectal bleeding, often with mucus, in an otherwise well-appearing infant [1]. In clinical practice, cow’s milk (CM) protein is the most common trigger, and diagnosis is largely based on clinical history, symptom resolution after dietary elimination, and recurrence after re-exposure, after exclusion of other causes of rectal bleeding [2,3,4]. Although FPIAP is generally considered a benign and self-limited condition, its clinical course is not uniform [4]. Recent cohort studies have shown that tolerance may be delayed in infants with immunoglobulin E (IgE) sensitization, multiple food allergies, and selected atopic features, particularly atopic dermatitis (AD) [5,6,7]. Current guidelines and real-world studies also indicate substantial heterogeneity in management, including the extent of maternal elimination, the choice of formula, and the timing of reintroduction. This variability is clinically relevant because unnecessary or prolonged dietary restriction may compromise breastfeeding and increase nutritional and psychosocial burden in a condition that often resolves during infancy [3,8,9,10].

Existing studies have primarily focused on patient-related predictors of delayed tolerance, whereas the contribution of management-related factors has remained less well defined [5,6,7,11,12]. In particular, it is unclear whether practices such as broader elimination diets, elimination-first approaches, or delayed reintroduction simply reflect perceived disease severity or are themselves associated with later tolerance acquisition. Clarifying this distinction is important for clinical care, as it may help identify potentially modifiable management strategies that are associated with a more prolonged disease course.

In this retrospective cohort study, we primarily investigated whether management-related factors, especially the initial management strategy, the extent of elimination, and the time to reintroduction, were associated with the timing of tolerance acquisition in infants with CM-induced FPIAP. Additionally, the secondary aim was to describe the clinical course and real-world management patterns of this condition in routine pediatric allergy practice.

## 2. Materials and Methods

### 2.1. Study Design

This retrospective cohort study was conducted across pediatric allergy outpatient clinics at tertiary referral centers. Infants evaluated between January 2021 and December 2025 with a diagnosis of CM-induced FPIAP were screened for eligibility. The study was conducted in accordance with the Declaration of Helsinki and received approval from the local ethics committee. The ethics committee waived the requirement for informed consent from participants for this study, given its retrospective, non-interventional design, and all data were anonymized before analysis.

### 2.2. Study Population

Infants were eligible if they had a clinically documented CM-induced FPIAP diagnosed during routine care by the treating pediatric allergist, had clinical features consistent with current guideline descriptions [4,9], and had alternative causes of rectal bleeding excluded. Infants were excluded if an alternative etiology for rectal bleeding was identified, including infectious colitis, necrotizing enterocolitis, Hirschsprung disease or Hirschsprung-associated enterocolitis, inflammatory bowel disease, coagulation disorders, isolated anal fissure without associated gastrointestinal symptoms, or other anatomic or surgical gastrointestinal conditions. Infants with follow-up less than 3 months or with insufficient documentation to support a clinically compatible diagnosis were also excluded.

A total of 362 medical records were screened, and 180 infants met eligibility criteria and were included in the final analysis. A detailed flowchart of study population selection, including reasons for exclusion, is provided in Figure S1.

### 2.3. Data Collection

Clinical data were retrospectively extracted from electronic medical records using a standardized abstraction form. Baseline variables included sex, age at symptom onset, symptom duration before specialist assessment, feeding modality at presentation, and follow-up duration. Presenting symptoms included rectal bleeding, mucus in the stool, irritability or feeding-related discomfort, diarrhea, and failure to thrive (FTT). Atopic background variables included physician-diagnosed AD and first-degree family history of atopy. Laboratory investigations and allergy testing were not performed systematically and were documented only when obtained during routine clinical care, at the physician’s discretion. Available laboratory data included hemoglobin (Hb), absolute eosinophil count, and total IgE. IgE sensitization to food allergens was assessed by skin prick testing (SPT) and/or by serum-specific IgE (sIgE) measurement. A positive SPT was defined as a wheal diameter at least 3 mm greater than the negative control, and detectable serum-specific IgE as ≥0.35 kU/L, as described in guidelines [13]. Peripheral eosinophilia was defined as >500 cells/µL, and anemia as Hb < 10.5 g/dL. Anemia was further categorized as mild (9.5–10.4 g/dL), moderate (7.0–9.4 g/dL), or severe (<7.0 g/dL) [14].

### 2.4. Management Strategies

Initial management was categorized based on the physician’s decision at the initial visit, as documented in the medical record, as either an observation-first or an elimination-first approach. Observation-first management was defined as an initial period of clinical observation without immediate dietary intervention. This approach was used in selected clinically stable infants, generally those with mild symptoms, preserved growth, and no immediate concern for systemic involvement or nutritional compromise. Because this was a retrospective real-world cohort, the observation period was not defined by a fixed study protocol. The duration of observation and the decision to initiate dietary elimination were determined by the treating physician based on symptom persistence, worsening rectal bleeding or mucus, feeding-related discomfort, inadequate weight gain, parental concern, or other clinical concerns during follow-up. Elimination-first management was defined as immediate removal of CM protein after diagnosis, by maternal CM elimination in breastfed infants and/or by replacing standard formula with a hypoallergenic formula in formula-fed or mixed-fed infants. Maternal elimination was further classified as CM elimination only or multi-food elimination. Multi-food elimination was defined as eliminating CM along with at least one additional suspected food trigger. When formula treatment was used, the type of hypoallergenic formula was also recorded as extensively hydrolyzed (eHF), amino acid (AA), or hydrolyzed rice formula (HRF), with preferences based on market availability.

### 2.5. Dietary Escalation, Reintroduction, and Outcome Definitions

Definitions were based on information documented in the medical records during routine clinical follow-up. Dietary escalation was defined as any intensification of dietary elimination after the initial management approach. Reintroduction was defined as any documented re-exposure to CM protein after a period of elimination, whether planned or unplanned, including hospital-based supervised reintroduction, home-based reintroduction, and unintentional exposure followed by continued feeding. The planned reintroduction was individualized based on symptom resolution, clinical stability, and physician judgment, rather than a uniform, prespecified schedule. Relapse was defined as recurrence of rectal bleeding and/or other symptoms consistent with FPIAP after re-exposure. Tolerance was defined as the absence of symptom recurrence during continued age-appropriate exposure to CM protein during follow-up. Time to tolerance was defined as the interval from diagnosis to documented tolerance. Tolerance was further categorized as early if achieved before 12 months of age, and delayed if achieved at or after 12 months of age.

### 2.6. Statistical Analyses

All statistical analyses were performed using Jamovi (version 2.3.26, The Jamovi Project, Sydney, Australia). Graphs and figures were generated using GraphPad Prism (version 9.5.1 for macOS, GraphPad Software, Boston, MA, USA) and Adobe Illustrator 2023 (version 27.9.0 for macOS, Adobe Inc., San Jose, CA, USA). Continuous variables were summarized as mean ± standard deviation (SD), or median with interquartile range (IQR), as appropriate, and categorical variables were summarized as frequencies and percentages. Analyses were performed using available data, and denominators were reported where data were missing.

Comparisons between categorical variables were performed using the Pearson χ^2^ test or Fisher’s exact test, as appropriate. Continuous variables were compared using Student’s *t* test or the Mann–Whitney U test, depending on distributional assumptions. Exploratory subgroup analyses were performed using descriptive statistics and the Kruskal–Wallis test. Binary logistic regression analysis was used to examine associations between candidate variables and binary outcomes, and multivariable logistic regression models were constructed to estimate adjusted odds ratios (ORs) with 95% confidence intervals (CIs). Overall model fit was assessed using likelihood-ratio statistics and McFadden’s pseudo-R^2^. Time-to-event analyses were performed using the Kaplan–Meier method, and survival curves were compared using the log-rank test. Cox proportional hazards regression was used to estimate hazard ratios (HRs) with 95% confidence intervals. Infants without documented tolerance, including those lost to follow-up, were censored at their last available follow-up in time-to-event analyses. The proportional hazards assumption for Cox regression models was assessed using Schoenfeld residual testing. To improve the reliability of delayed tolerance classification, a landmark-type sensitivity analysis was performed by restricting the analysis to infants with follow-up duration ≥ 12 months. Additional sensitivity analyses were performed using available clinical proxies of disease severity and by excluding infants with confirmed multiple food allergies. Because of the reduced sample size in this subgroup, a parsimonious logistic regression model was used. All tests were two-sided, and *p*-values < 0.05 were considered statistically significant.

## 3. Results

### 3.1. Baseline Characteristics of the Study Cohort

A total of 180 infants with CM-induced allergic proctocolitis were included. The cohort comprised 53.3% *(n* = 96) males and 46.7% (*n* = 84) females. The mean age at symptom onset was 6.8 ± 2.2 weeks, and the median age at diagnosis was 8.0 weeks (IQR, 7–10). The median follow-up duration was 9.5 months (IQR, 6–14).

**Figure 1:**
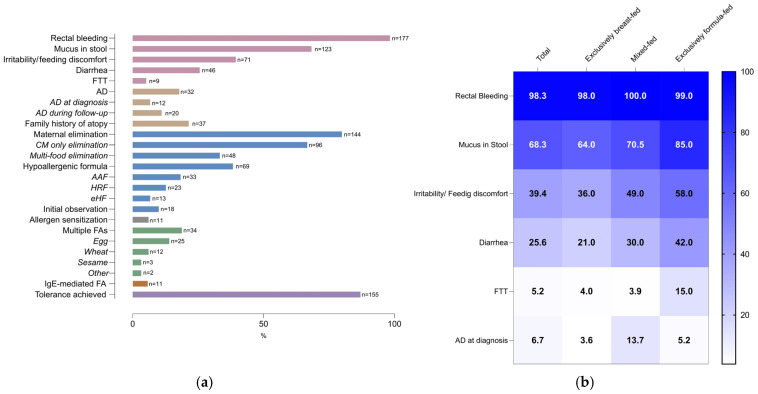
Clinical characteristics, management strategies, and outcomes in infants with cow’s milk protein-induced allergic proctocolitis. (**a**) Overall frequency (%) of presenting symptoms, atopic and family history variables, initial management patterns, formula types, sensitization findings, and tolerance achievement in the total cohort. The numbers shown next to the bars indicate the sample sizes, and the italicized labels denote subgroups nested within the corresponding main category. (**b**) Heatmap showing the frequency (%) of selected clinical features at presentation according to feeding type (total, exclusively breast-fed, mixed-fed, and exclusively formula-fed). Color intensity reflects the proportion of patients in each category. (AAF, amino acid-based formula; AD, atopic dermatitis; CM, cow’s milk; eHF, extensively hydrolyzed formula).

At presentation, 61.1% (*n* = 110) of infants were exclusively breast-fed, 28.3% (*n* = 51) were mixed-fed, and 10.6% (*n* = 19) were exclusively formula-fed. Rectal bleeding was the predominant presenting symptom (98.3%), followed by mucus in stool (68.3%), irritability/feeding discomfort (39.4%), and diarrhea (25.6%); failure to thrive was uncommon (5.2%). Atopic dermatitis was observed in 17.8% of infants during follow-up, and 21.4% had a family history of atopy (Figure 1a).

There were no statistically significant differences in the frequencies of rectal bleeding, mucus in stool, diarrhea, irritability/feeding discomfort, or atopic dermatitis across feeding modalities (all *p* > 0.05). Although symptoms other than rectal bleeding were numerically more frequent among exclusively formula-fed infants, these between-group differences did not reach statistical significance (Figure 1b). Laboratory evaluation was performed in 33.9% (*n* = 61) of infants. Baseline clinical and laboratory characteristics of the cohort are summarized in Supplementary Table S1.

### 3.2. Initial Management Strategies and Symptom Resolution:

An observation-first approach was used in 10.0% (*n* = 18) of infants, whereas an elimination diet-based approach was used initially in 90.0% (*n* = 162), including maternal elimination alone (51.7%, *n* = 93), maternal elimination combined with hypoallergenic formula (28.3%, *n* = 51), and hypoallergenic formula alone (10.0%, *n* = 18). Overall, maternal elimination was implemented in 80.0% (*n* = 144) of infants; among these, 66.7% (*n* = 96) received CM elimination only, and 33.3% (*n* = 48) underwent multi-food elimination. Hypoallergenic formula was used in 38.3% (*n* = 69/180) of infants, including AAF in 47.8% (*n* = 33/69), eHF in 18.8% (*n* = 13/69), and HRF in 33.3% (*n* = 23/69). Among infants initially managed with observation-first, complete symptom resolution without dietary intervention occurred in 44.4% (*n* = 8/18). Elimination was subsequently initiated in 55.6% (*n* = 10/18), including 22.2% (*n* = 4/18) due to severe irritability after 12–15 days of observation and 33.3% (*n* = 6/18) due to parental preference despite no documented clinical worsening. During follow-up, dietary escalation was recorded in 30.9% (*n* = 50/162) of infants, at a median of 7 weeks (IQR, 4–12) after the initial intervention. Of these, 20.0% (*n* = 10/50) had initially been managed with observation-first and 80.0% (*n* = 40/50) with elimination-first.

Changes in feeding practices and formula use were also documented during follow-up. Among infants who were exclusively breast-fed at presentation, formula supplementation was introduced in 8 of 110 (7.3%), of whom 6 subsequently discontinued breastfeeding. Among infants receiving mixed feeding at presentation, 9 of 51 (17.6%) discontinued breastfeeding and switched to exclusive formula feeding. Overall, breastfeeding cessation occurred in 17 of 161 infants receiving any breastfeeding at presentation (10.6%). Reported reasons included perceived low milk supply in 9 of 17 infants (52.9%), breast refusal in 4 of 17 (23.5%), and unwillingness to continue maternal dietary restriction in 4 of 17 (23.5%). Among infants receiving hypoallergenic formula, 10 of 33 infants initially receiving AAF switched to HRF (30.3%), mainly due to palatability issues or formula refusal. Conversely, 6 of 23 infants initially receiving HRF switched to AAF (26.1%), mainly due to inadequate weight gain.

Loss to follow-up occurred in 10 infants (5.6%), all of whom were initially managed with an elimination-first approach. Baseline characteristics of infants with available outcome data and those lost to follow-up are presented in Supplementary Table S2. Overall, no major baseline differences were observed between groups, except that those infants lost to follow-up had a lower age at diagnosis than those with available outcome data (median, 7.25 vs. 8.0 weeks; Mann–Whitney *p* = 0.023). Reintroduction was attempted in 162 of 170 evaluable infants (95.3%) at a median age of 8.5 months (IQR, 7.0–10.5), corresponding to a median interval of 28.1 weeks (IQR, 23.8–33.4) from diagnosis. Reintroduction was not attempted in 8 infants, all of whom had achieved symptom resolution with an observation-first approach. Relapse after reintroduction occurred in 31 of 162 infants (19.1%). Among the factors evaluated for association with relapse, only the interval from diagnosis to reintroduction was statistically significant. Infants who relapsed had a significantly longer interval from diagnosis to reintroduction than those who did not relapse (*p* = 0.03). Consistently, time-to-event analysis showed that delayed reintroduction was associated with a higher probability of relapse, with a statistically significant difference by the log-rank test (*p* = 0.043). Reintroduction was not attempted in 8 infants, all of whom had achieved symptom resolution with an observation-first approach.

Tolerance was documented in 155 of 170 infants (91.2%), with a median time from diagnosis to tolerance of 31.1 weeks (IQR, 26.1–38.2) and a median documented duration of continued exposure of 12 weeks (IQR, 8–20). Among them, 124 infants (80.0%) achieved early tolerance and 31 (20.0%) had delayed tolerance. Multiple concurrent food allergies were identified in 34 of 170 infants (19.4%). Of those who underwent multi-food elimination at presentation, 16 of 48 (33.3%) were eventually diagnosed with multiple food allergy, whereas 32 of 48 (66.7%) were not. During follow-up, IgE-mediated food allergy was documented in 11 of 170 infants (6.5%). Baseline CM sensitization testing was available in 4 of these 11 infants, and 2 were positive.

Initial management, follow-up interventions, and outcomes in infants with CM-induced FPIAP are presented in Table 1.

### 3.3. Factors Associated with Tolerance Development in Infants with CM-Induced FPIAP

**Figure 2:**
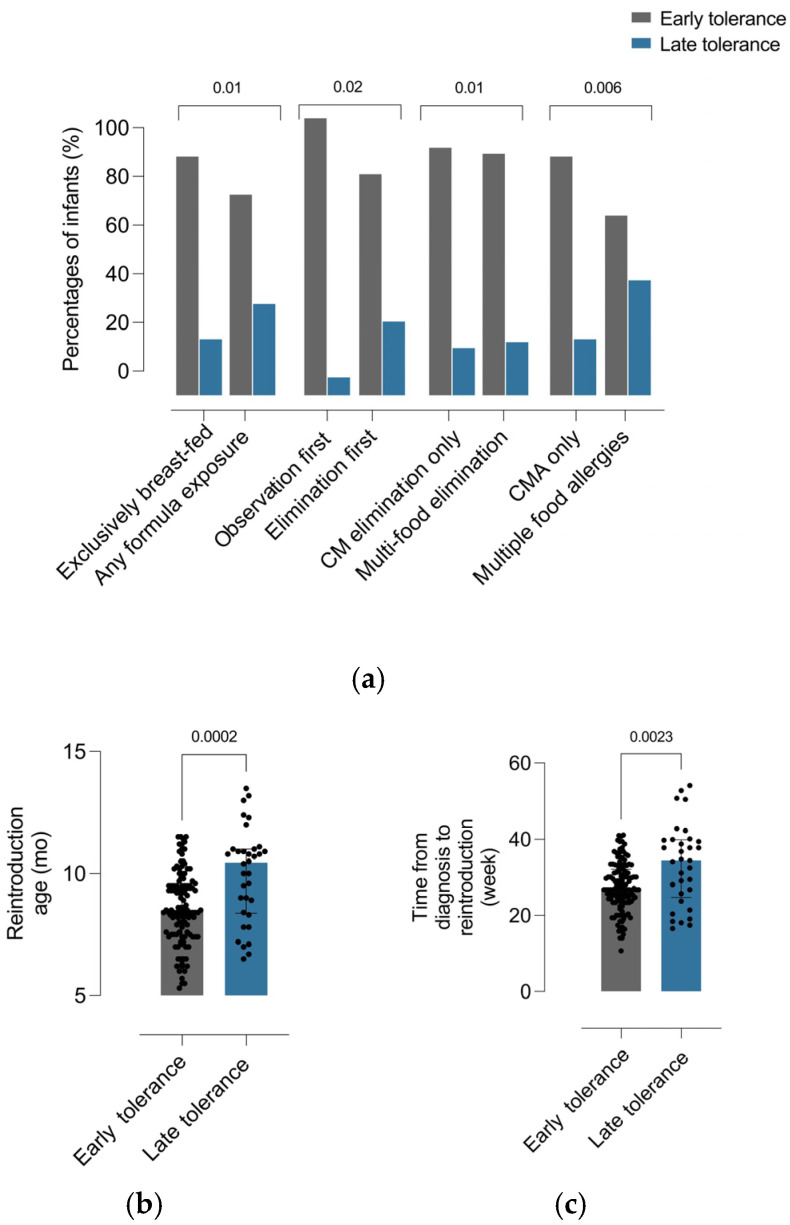
Clinical correlates of early versus late tolerance acquisition in infants with cow’s milk protein-induced allergic proctocolitis. (**a**) Distribution of clinical and management characteristics according to tolerance phenotype, including feeding exposure, initial management strategy, elimination pattern, and allergy phenotype. Data are presented as percentages, and categorical variables were compared using the χ^2^ test or Fisher’s exact test, as appropriate. (**b**) Age at reintroduction according to tolerance phenotype. (**c**) Time from diagnosis to reintroduction according to tolerance phenotype. In panels (**b**,**c**), each dot represents an individual infant. Continuous variables are presented as median (IQR) and were compared using the Mann–Whitney *U* test. Exact *p*-values are shown above the corresponding comparisons, and a *p*-value < 0.05 was considered statistically significant (CM, cow’s milk; CMA, cow’s milk allergy; IQR, interquartile range; mo, months; wk, weeks).

In comparisons between infants with early and late tolerance, exclusive breastfeeding and observation-first management were more frequent in the early tolerance group (*p* = 0.04 and *p* = 0.02, respectively), whereas multi-food elimination and concomitant multiple food allergies were significantly more frequent in the late tolerance group (*p* = 0.01 and *p* = 0.006) (Figure 2a). In addition, infants with late tolerance had a significantly higher age at reintroduction (*p* = 0.0002) (Figure 2b) and a longer interval from diagnosis to reintroduction (*p* = 0.0023) (Figure 2c).

In univariable logistic regression analyses, exclusive breastfeeding was associated with lower odds of delayed tolerance (OR, 0.48; 95% CI, 0.24–0.98; *p* = 0.044). In contrast, multi-food elimination at presentation (OR, 2.97; 95% CI, 1.25–7.02; *p* = 0.013), concomitant multiple food allergies (OR, 2.65; 95% CI, 1.32–6.57; *p* = 0.008), and a longer interval from diagnosis to reintroduction (OR per week, 1.10; 95% CI, 1.04–1.16; *p* = 0.002) were associated with higher odds of delayed tolerance. Diet escalation showed a borderline association (OR, 2.23; 95% CI, 0.95–5.19; *p* = 0.062). Other variables, including irritability or feeding discomfort, diarrhea, FTT, AD, and observation-first management, were not significantly associated, although observation-first management showed a trend toward lower odds (OR, 0.18; 95% CI, 0.02–1.38; *p* = 0.098). In multivariable analysis, only multi-food elimination at presentation (OR, 2.58; 95% CI, 1.02–6.54; *p* = 0.046) and the interval from diagnosis to reintroduction (OR per week, 1.08; 95% CI, 1.02–1.14; *p* = 0.022) remained independently associated with delayed tolerance. Exclusive breastfeeding and concomitant multiple food allergies were no longer significant after adjustment (OR, 0.92; 95% CI, 0.36–2.34; *p* = 0.921; and OR, 1.85; 95% CI, 0.73–4.71; *p* = 0.194, respectively). The overall model was significant (χ^2^ = 15.0, df = 4, *p* = 0.005; McFadden R^2^ = 0.111) (Table 2).

To assess the robustness of the main findings, a sensitivity analysis was performed after excluding infants with confirmed multiple food allergies. In the adjusted model within this restricted subgroup, the direction of the main associations remained consistent with the primary analysis. Multi-food elimination at presentation, dietary escalation, and a longer interval from diagnosis to reintroduction were associated with delayed tolerance, whereas exclusive breastfeeding did not remain independently associated with delayed tolerance after adjustment (Supplementary Table S3).

Dietary escalation was also evaluated separately as an exploratory marker of persistent or worsening symptoms during follow-up. In time-to-event analysis, dietary escalation was not significantly associated with time to CM tolerance, although infants requiring escalation showed a numerically lower hazard of tolerance acquisition (HR, 0.65; 95% CI, 0.22–1.88; *p* = 0.428). Relapse after CM reintroduction did not differ significantly by dietary escalation status among infants with available data for both variables (*p* = 0.655). Thus, dietary escalation was associated with delayed tolerance in the restricted logistic model but was not significantly associated with time-to-tolerance or relapse in exploratory analyses.

Among infants on hypoallergenic formula, median time to tolerance was 32.8 weeks (IQR 26.8–37.4) in the eHF group, 31.1 weeks (IQR 28.9–36.8) in the AAF group, and 37.4 weeks (IQR 25.3–46.5) in the HRF group. No statistically significant difference was observed across formula types (Kruskal–Wallis χ^2^ = 1.31, df = 2, *p* = 0.518).

To enhance the accuracy of delayed tolerance assessment, a landmark sensitivity analysis was conducted among infants with a follow-up duration of ≥12 months. This subgroup included 60 infants, of whom 43 (71.7%) achieved early tolerance, and 17 (28.3%) had delayed tolerance. Because of the reduced sample size and the limited number of delayed tolerance events, a parsimonious logistic regression model was used, including multi-food elimination at presentation and time from diagnosis to reintroduction. The direction of the associations was consistent with the primary analysis, although statistical significance was attenuated. Multi-food elimination showed numerically higher odds of delayed tolerance (OR, 2.07; 95% CI, 0.52–8.26; *p* = 0.301), and longer time from diagnosis to reintroduction suggested a similar trend (OR per week, 1.07; 95% CI, 0.98–1.18; *p* = 0.115).

**Figure 3:**
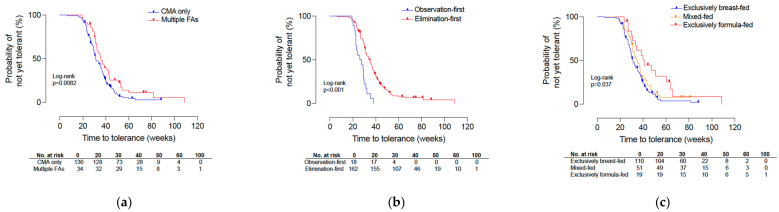
Kaplan–Meier analysis of time to tolerance in infants with cow’s milk protein-induced allergic proctocolitis. The y-axis indicates the probability of not yet being tolerant. (**a**) Time to tolerance according to allergy phenotype, comparing infants with isolated cow’s milk allergy and those with multiple food allergies. (**b**) Time to tolerance according to initial management strategy, comparing observation-first and elimination-first management. (**c**) Time to tolerance according to feeding modality at presentation, comparing exclusively breast-fed, mixed-fed, and exclusively formula-fed infants. Time to tolerance is expressed in weeks. Group comparisons were performed using the log-rank test, and *p*-values are shown within each panel. Numbers below each panel indicate the number of infants at risk at the corresponding time points, defined as infants who had not yet achieved tolerance and had not been censored. Panel (**a**) was restricted to infants with available allergy phenotype and outcome data (*n* = 170). In Panels (**b**,**c**), infants lost to follow-up were included and censored at their last available follow-up. A *p*-value < 0.05 was considered statistically significant. CMA, cow’s milk allergy; FA, food allergy.

Kaplan–Meier analysis demonstrated that infants with concomitant multiple food allergies had a longer time to tolerance than those with only CM allergy (log-rank *p* = 0.0082), with median times to tolerance of 34.4 weeks (95% CI, 32.1–42.1) and 30.1 weeks (95% CI, 29.1–33.4), respectively. In univariable Cox regression, concomitant multiple food allergies were associated with a lower hazard of tolerance acquisition (HR, 0.60; 95% CI, 0.41–0.88; *p* = 0.009) (Figure 3a). Time to tolerance also differed according to initial management strategy (log-rank *p* < 0.001). Median time to tolerance was 26.9 weeks (95% CI, 22.8–31.4) in the observation-first group and 32.1 weeks (95% CI, 30.1–35.4) in the elimination-first group. In univariable Cox regression, elimination-first management was associated with a lower hazard of tolerance acquisition than observation-first management (HR, 0.37; 95% CI, 0.22–0.62; *p* < 0.001) (Figure 3b). Time to tolerance differed according to feeding modality at presentation (log-rank *p* = 0.037). Median time to tolerance was 30.1 weeks (95% CI, 29.1–33.4) in exclusively breast-fed infants, 32.1 weeks (95% CI, 30.1–36.8) in mixed-fed infants, and 34.9 weeks (95% CI, 30.1–60.2) in exclusively formula-fed infants. In univariable Cox regression, exclusively formula-fed infants had a significantly lower hazard of tolerance acquisition than exclusively breastfed infants (HR, 0.48; 95% CI, 0.27–0.86; *p* = 0.013), whereas mixed-fed infants did not differ significantly from exclusively breastfed infants (HR, 0.89; 95% CI, 0.62–1.28; *p* = 0.533) (Figure 3c). Schoenfeld residual testing did not indicate violation of the proportional hazards assumption in the Cox models evaluating observation-first versus elimination-first management (global χ^2^ = 0.328, df = 1, *p* = 0.567), multi-food elimination at presentation (global χ^2^ = 0.254, df = 1, *p* = 0.614), or feeding modality at presentation (global χ^2^ = 1.765, df = 2, *p* = 0.414).

## 4. Discussion

In this large, well-characterized cohort of infants with CM-induced FPIAP, the overall clinical course was favorable, with most infants achieving tolerance within infancy. This observation is consistent with previous reports and guideline documents describing FPIAP as a generally transient, non-IgE-mediated food allergy [1,2,3,4,8,15]. Beyond confirming the expected natural history, our data provide further insight into factors associated with the timing of tolerance acquisition. A key finding of our study was the association between delayed reintroduction and later tolerance acquisition. In multivariable analysis, each additional week before reintroduction was associated with an 8% increase in the odds of delayed tolerance. From a clinical perspective, even relatively short delays in reintroduction may cumulatively contribute to a meaningful postponement of tolerance development. This observation is in line with recent literature that has increasingly questioned prolonged elimination strategies and supported earlier reintroduction once symptoms have resolved [4,7,9,10,11,16]. Taken together, these findings raise the possibility that delayed reintroduction may not be biologically neutral and may be associated with delayed tolerance acquisition.

Multi-food elimination at presentation also remained independently associated with delayed tolerance. This observation is clinically relevant because broad maternal elimination is frequently used in routine practice despite limited evidence that it improves outcomes in uncomplicated FPIAP [9,10,16]. Notably, most infants who underwent multi-food elimination at presentation were not ultimately classified as having multiple food allergies, indicating that the extent of dietary restriction may, in some cases, exceed the eventual allergy phenotype. Broader dietary restriction may also plausibly reduce antigen exposure during a period relevant to tolerance development, in line with current mechanistic concepts of oral tolerance [17,18,19,20]. Importantly, the association between multi-food elimination and delayed tolerance remained consistent in the sensitivity analysis excluding infants with confirmed multiple food allergies, suggesting that this finding was not solely driven by infants with broader allergic phenotypes. Nevertheless, residual confounding by clinical severity and physician decision-making cannot be excluded.

Infants with multiple food allergies also appeared to have a different clinical trajectory. While the association with delayed tolerance did not persist in multivariable analysis, time-to-event analysis demonstrated a lower probability of achieving tolerance over time in this group. This finding aligns with previous studies suggesting that more complex or broader allergic phenotypes tend to resolve more slowly [5,6,7].

In our cohort, exclusive breastfeeding was associated with a lower likelihood of delayed tolerance in univariable analysis; however, this association did not remain significant after multivariable adjustment, in keeping with previous reports [7]. In contrast, Kaplan–Meier analysis demonstrated earlier tolerance acquisition among exclusively breastfed infants than among those receiving mixed or formula feeding. This pattern is broadly consistent with studies suggesting that dietary factors, including maternal diet, may influence the timing of tolerance development in FPIAP [11].

Although observation-first management was not independently associated with delayed tolerance after multivariable adjustment, Kaplan–Meier analysis showed earlier tolerance acquisition in this group than in infants managed with an elimination-first strategy. This difference likely reflects the complementary nature of the analytical approaches rather than a true inconsistency, as logistic regression categorizes the outcome as early versus delayed tolerance, whereas survival analysis captures the timing of tolerance acquisition across the entire follow-up period. In this context, the shorter time to tolerance observed in the observation-first group suggests that immediate dietary restriction may not always be necessary in selected infants at first presentation, in keeping with previous reports describing spontaneous resolution without prompt elimination [21,22,23,24]. However, this finding should be interpreted cautiously because observation-first management was used in a small subgroup and was not randomly assigned. In routine practice, infants selected for observation-first management may have had milder symptoms or lower clinical concern at presentation, raising the possibility of confounding by indication. Therefore, the observed association between observation-first management and earlier tolerance acquisition should be considered exploratory and should not be interpreted as evidence of a causal benefit. Propensity score-based adjustment was not performed because the small size of the observation-first group limited covariate overlap and would likely have produced unstable estimates.

Dietary escalation was associated with delayed tolerance in the restricted logistic model but was not significantly associated with time-to-tolerance or relapse in exploratory analyses. This discrepancy likely reflects the role of dietary escalation as a marker of clinical complexity or persistent symptoms rather than a direct causal determinant of delayed tolerance. Considered together, these observations suggest that the course of FPIAP may be shaped not only by baseline clinical characteristics but also by management patterns over time. In particular, the observation-first subgroup illustrates that spontaneous resolution without immediate dietary restriction is possible in selected cases. Alongside the associations observed for delayed reintroduction and multi-food elimination, this supports a more individualized and less restrictive approach to management, especially in uncomplicated presentations [4,9].

The strengths of this study include a relatively large cohort, a real-world design, and a detailed characterization of management practices during follow-up. In addition, the combined use of descriptive comparisons, logistic regression, and time-to-event analyses enabled examining whether tolerance developed and how its timing varied across clinical and management-related factors. However, several limitations should be considered when interpreting these findings. First, the retrospective design limited the standardization of diagnosis, management decisions, reintroduction practices, and follow-up. Outcome ascertainment relied on medical record documentation rather than a predefined prospective protocol. In addition, management strategies were determined by treating physicians and families in routine practice, which may have introduced confounding by indication, particularly for the observation-first subgroup. Although sensitivity analyses using available clinical proxies of disease severity were performed, residual confounding cannot be excluded because no standardized symptom severity score was available. Second, several subgroup analyses were limited by small sample sizes, including those involving observation-first management, formula-fed infants, formula-type comparisons, IgE sensitization, and the landmark-type sensitivity analysis, which was restricted to infants with follow-up duration ≥12 months. Therefore, these findings should be interpreted as supportive and exploratory rather than confirmatory. The multivariable model was intentionally kept parsimonious because of the limited number of delayed tolerance events. Several potentially relevant confounders, including standardized symptom severity, maternal dietary adherence, probiotic exposure, and detailed dietary restriction intensity, could not be fully incorporated because they were either not systematically recorded or were available only in selected patients. Third, although multi-food elimination was defined as CM elimination plus at least one additional suspected food trigger, the exact number of eliminated foods per infant was not consistently available. Therefore, the intensity of dietary restriction within the multi-food elimination subgroup could not be further quantified. Similarly, laboratory data were available for only a subset of infants, as testing was obtained according to routine clinical judgment rather than a standardized protocol. This may have introduced selection bias, since infants with more persistent, severe, or clinically complex symptoms may have been more likely to undergo laboratory evaluation. Consequently, immune markers such as eosinophil counts, total IgE, and IgE sensitization could not be reliably incorporated into the primary prognostic models, and their interpretation should be considered exploratory. Accordingly, the associations identified in this study should be interpreted as observations from real-world clinical practice rather than causal effects, and they require confirmation in prospective studies with standardized management pathways, systematic laboratory assessment, and predefined follow-up schedules.

## 5. Conclusions

CM-induced FPIAP is generally a self-limited condition. However, the timing of tolerance acquisition was heterogeneous and was associated with both clinical phenotype and management-related factors. Based on our data, delayed reintroduction and multi-food elimination were confirmed to be associated with later tolerance. These observations support a cautious and individualized approach to management for FPIAP patients starting at initial presentation and during their follow-up.

## Figures and Tables

**Table 1:** Initial management, follow-up interventions, and outcomes in infants with cow’s milk protein-induced allergic proctocolitis.

Characteristic	Value *
Initial management	
Initial approach, *n* (%)	180 (100)
The observation-first approach	18 (10.0)
An elimination diet-based approach	162 (90.0)
Maternal elimination diet	93 (51.7)
Maternal elimination diet with hypoallergenic formula	51 (28.3)
Hypoallergenic formula only	18 (10.0)
Maternal elimination, *n*/*N* (%)	144/180 (80.0)
Cow’s milk elimination only	96/144 (66.7)
Multi-food elimination	48/144 (33.3)
Hypoallergenic formula type, *n*/*N* (%)	69/180 (38.3)
Amino acid formula (AAF)	33/69 (47.8)
Extensively hydrolysed formula (eHF)	13/69 (18.8)
Hydrolysed rice formula (HRF)	23/69 (33.3)
Observation-first subgroup, *n*/*N* (%)	18/180 (10.0)
Complete resolution without dietary intervention	8/18 (44.4)
Switched to elimination after observation	10/18 (55.6)
Follow-up management and outcome	
Switched from AAF to HRF, *n*/*N* (%)	10/33 (30.3)
Switched from HRF to AAF, *n*/*N* (%)	6/23 (26.1)
Switched from breastfeeding to formula feeding, *n*/*N* (%)	6/110 (5.5)
Switched from breastfeeding to mixed feeding, *n*/*N* (%)	2/110 (1.8)
Switched from mixed feeding to formula feeding, *n*/*N* (%)	9/51 (17.6)
Breastfeeding cessation, *n*/*N* (%)	17/161 (10.5)
^†^ Lost to follow-up *n*/*N* (%)	10/180 (5.6)
Diet escalation, *n*/*N* (%)	50/162 (30.9)
Reintroduction attempted, *n*/*N* (%)	162/170 (95.3)
Age at reintroduction, months, median (IQR)	8.5 (7.0–10.5)
Time from diagnosis to reintroduction, wks, median (IQR)	28.1 (23.8–33.4)
Relapse after reintroduction, *n*/*N* (%)	31/162 (19.1)
Tolerance achieved, *n*/*N* (%)	155/170 (91.2)
Time from diagnosis to tolerance, wks, median (IQR)	31.1 (26.1–38.2)
Multiple concomitant FAs, *n*/*N* (%)	34/170 (19.4)
IgE-mediated FA during follow-up	11/170 (6.5)

FA, food allergy; IQR, interquartile range; *n*, subgroup count; *N*, total group count, wks; weeks. Data are presented as *n* (%), *n*/*N* (%), or median (IQR), as appropriate. * Percentages are calculated within the relevant subgroup denominator. ^†^ All patients lost to follow-up were on the elimination diet.

**Table 2:** Univariate and multivariable logistic regression analyses of factors associated with late tolerance (≥12 months).

Variable	Univariate OR	95% CI	*p*	Multivariable OR	95% CI	*p*
Exclusive breastfeeding	0.48	0.24–0.98	**0.044**	0.92	0.36–2.34	0.921
Irritability/feeding discomfort	0.62	0.20–1.33	0.219			
Diarrhea	1.63	0.71–3.74	0.247			
Failure to thrive	1.66	0.36–8.9	0.559			
Atopic dermatitis	1.94	0.79–4.78	0.147			
Observation-first management	0.18	0.02–1.38	0.098			
Multi-food elimination at presentation	2.97	1.25–7.02	**0.013**	2.58	1.02–6.54	**0.046**
Diet escalation	2.23	0.95–5.19	0.062			
Concomitant multiple food allergies	2.65	1.32–6.57	**0.008**	1.85	0.73–4.71	0.194
Time from diagnosis to reintroduction, weeks	1.10	1.04–1.16	**0.002**	1.08	1.02–1.14	**0.022**

Outcome was defined as delayed tolerance versus early tolerance. For continuous variables, odds ratios represent the change associated with a 1-week increase. Overall multivariable model: χ^2^ = 15.0, df = 4, *p* = 0.005; McFadden R^2^ = 0.111. (CI, confidence interval; OR, odds ratio). Bold values indicate statistically significant *p* values, defined as *p* < 0.05.

## Data Availability

The data that support the findings of this study are available from the corresponding author upon reasonable request.

## Supplementary Materials

The following supporting information can be downloaded at: https://www.mdpi.com/article/10.3390/jcm15103862/s1, Figure S1: Flowchart of study population selection, Table S1: Baseline characteristics of infants with cow’s milk protein-induced allergic proctocolitis. Table S2: Baseline characteristics of infants with available outcome data and those lost to follow-up. Table S3: Sensitivity analysis excluding infants with confirmed multiple food allergies.
