# Supplementary material for: Are Management Strategies Associated with Tolerance Acquisition in Infants with Cow’s Milk-Induced Allergic Proctocolitis?
Source: J Clin Med. 2026 May 17;15(10):3862. doi: 10.3390/jcm15103862 (PMC13207369; doi:10.3390/jcm15103862)
Supplement: Supplementary file 1 [file jcm-15-03862-s001.zip › Supplementary Materials.pdf]

Supplementary Materials

Baseline laboratory characteristics of *CM-induced FPIAP*

Laboratory evaluation was performed in 33.9% (n=61) of infants. Among those evaluated, median Hb was 11.6 g/dL (IQR, 10.9-12.1), absolute eosinophil count was 250 cells/μL (IQR, 210-680), and total IgE was 20 IU/mL (IQR, 12.5-104). Peripheral eosinophilia was present in 16.1% (n=10). Mild anemia was present in 26.2% (n=16/61) of infants, whereas moderate anemia was observed in 8.2% (n=5/61). Among infants who underwent laboratory evaluation, those with AD (n=15/61) had higher eosinophil counts (median, 478 [IQR, 412-610] vs. 273 [IQR, 195-390] cells/μL; p=0.003) and higher total IgE levels (median, 80.2 [IQR,30.4-120.5] vs. 18.0 [IQR, 10-24] IU/mL; p <0.001) than those without AD (n=46/61). IgE sensitization to food allergens was assessed in 20.5% of infants (n=37), including 9.4% (n=17) at baseline and 11.1% (n=20) during follow-up. The most common indication for assessment was AD, observed in 59.5% (n = 22/37) of infants, followed by atypical symptoms such as poor weight gain or vomiting in 18.9% (n = 7/37), and parental concern or request in 16.2% (n = 6/37); indication data were unavailable in 5.4% (n = 2/37). IgE sensitization to at least one food allergen was detected in 29.7% (n=11/37) of those tested, and 63.6% (n=7/11) of sensitized infants had concomitant AD. Cow's milk sensitization was present in 4 of 37 tested infants (10.8%), egg sensitization in 6 of 37 (16.2%), wheat sensitization in 2 of 37 (5.4%), sesame sensitization in 1 of 37 (2.7%), and sensitization to other foods in 1 of 37 (2.7%). Because sensitization testing was not performed systematically and some infants were sensitized to multiple foods, these findings were interpreted descriptively.

Supplementary Table S1: Baseline characteristics of infants with cow's milk protein–induced allergic proctocolitis.

Characteristic	Value*
**Demographic and clinical characteristics**	
Sex, n (%)	
Male	96 (53.3)
Female	84 (46.7)
Age at symptom onset, weeks, mean ± SD	6.8 ± 2.2
Age at diagnosis, weeks, median (IQR)	8.0 (7.0-10.0)
Feeding modality at presentation, n (%)	
Breastfeeding	110 (61.1)
Both breast and formula feeding (mixed)	51 (28.3)
Formula feeding	19 (10.6)
Presenting symptoms, n (%)	
Rectal bleeding	177 (98.3)
Mucus in stool	123 (68.3)
Irritability or feeding discomfort	71 (39.4)
Diarrhea	46 (25.6)
Failure to thrive	9 (5)
Atopic dermatitis, n (%)	32 (17.8)
Family history of atopy, n (%)	37 (21.4)
Follow-up duration, months, median (IQR)	9.5 (6.0-14.0)
**Laboratory findings**	
Laboratory evaluation performed n/N (%)	61/180 (33.9)
Hemoglobin (g/dL), median (IQR)	11.6 (10.9-12.1)
Absolute eosinophil count (cells/µL), median (IQR)	250 (210-680)
Total IgE (IU/mL), median (IQR)	20 (12.5-104)
**IgE-mediated sensitization**	
Sensitization assessed, n/N (%)	37/180 (20.5)
Sensitization to any food allergens, n/N (%)	11/37 (29.7%)
Cow's milk	4/37 (10.8%)
Egg	6/37 (16.2%)
Wheat	2/37 (5.4%)
Sesame	1/37 (2.7%)
Others	1/37 (2.7%)

IgE, immunoglobulin E; IQR, interquartile range; n, subgroup count; N, total group count; SD, standard deviation; SPT, skin prick test.

* Data are presented as n (%), mean ± SD, or median (IQR), as appropriate.

Supplementary Table S2: Baseline characteristics of infants with available outcome data and those lost to follow-up.

Variable	Infants with available outcome data (n=170)	Infants lost to follow-up, (n=10)	p value
Male sex, n/N (%)	89/170 (52.4)	7/10 (70.0)	0.34
Feeding modality at presentation, n/N (%)			0.78
Breastfeeding	103/170 (60.6)	7/10 (70.0)	
Both breast and formula feeding	48/170 (28.2)	3/10 (30.0)	
Formula feeding	19/170 (11.2)	0/10 (0.0)	
Age at symptom onset, weeks, mean± SD	7.0 ± 2.18	7.0 ± 2.07	0.66
Age at diagnosis, weeks, mean ± SD	8.0 ± 2.33	7.25 ± 1.68	**0.02**
Mucus in stool, n/N (%)	115/170 (67.6)	8/10 (80.0)	0.50
Diarrhea, n/N (%)	44/170 (25.9)	2/10 (20.0)	1.00
Failure to thrive, n/N (%)	9/164 (5.5)	0/9 (0.0)	1.00
Atopic dermatitis, n/N (%)	30/170 (17.6)	2/10 (20.0)	0.69
Family history of atopy, n/N (%)	34/163 (20.9)	3/10 (30.0)	0.44
Multi-food elimination at presentation, n/N (%)	43/134 (32.1)	5/10 (50.0)	0.30

Data are presented as n/N (%) or mean ± SD as appropriate. Fisher's exact test was used for categorical variables because of the small number of infants lost to follow-up. The Mann–Whitney U test was used for continuous variables.

Supplementary Table S3: Sensitivity analysis excluding infants with confirmed multiple food allergies.

Variable	Univariate OR	95% CI	p	Multivariable OR	95% CI	**p**
Exclusive breastfeeding	0.34	0.15–0.78	0.010	0.47	0.15–1.45	0.180
Irritability/feeding discomfort	1.30	0.58–2.92	0.530			
Diarrhea	1.21	0.48–3.06	0.68			
FTT	1.46	0.27–7.96	0.6600			
Atopic dermatitis	1.13	0.37–3.40	0.830			
Observation-first management	0.17	0.02–1.36	0.090			
Multi-food elimination at presentation	2.97	1.15–7.65	0.020	**3.29**	1.07–10.12	**0.030**
Diet escalation	2.86	1.13–7.22	0.020	**5.31**	1.57–17.93	**0.007**
Time from diagnosis to reintroduction, weeks	1.10	1.04–1.17	0.001	**1.09**	1.02–1.17	**0.015**

ORs indicate odds of delayed tolerance. The analysis was restricted to infants without confirmed multiple food allergies. Variables with p<0.05 in univariate analysis and clinically relevant variables were considered for the multivariable model. CI, confidence interval; OR, odds ratio.

Supplementary Figure Legends:

Figure S1. Flowchart of study population selection.

Of 362 screened medical records, 180 infants met eligibility criteria and were included in the final analysis. The remaining 182 infants were excluded because of insufficient documentation to support a guideline-compatible diagnosis of FPIAP at presentation (n=65), insufficient demographic or clinical data (n=27), follow-up shorter than 3 months (n=66), FPIAP triggered by foods other than cow's milk (n=22), or a subsequent diagnosis of inflammatory bowel disease during follow-up (n=2).

(FPIAP, food protein-induced allergic proctocolitis; IBD, inflammatory bowel disease.)
