# Supplementary figures and images for: Are Management Strategies Associated with Tolerance Acquisition in Infants with Cow’s Milk-Induced Allergic Proctocolitis?
Source: J Clin Med. 2026 May 17;15(10):3862. doi: 10.3390/jcm15103862 (PMC13207369; doi:10.3390/jcm15103862)

**Figure S1**

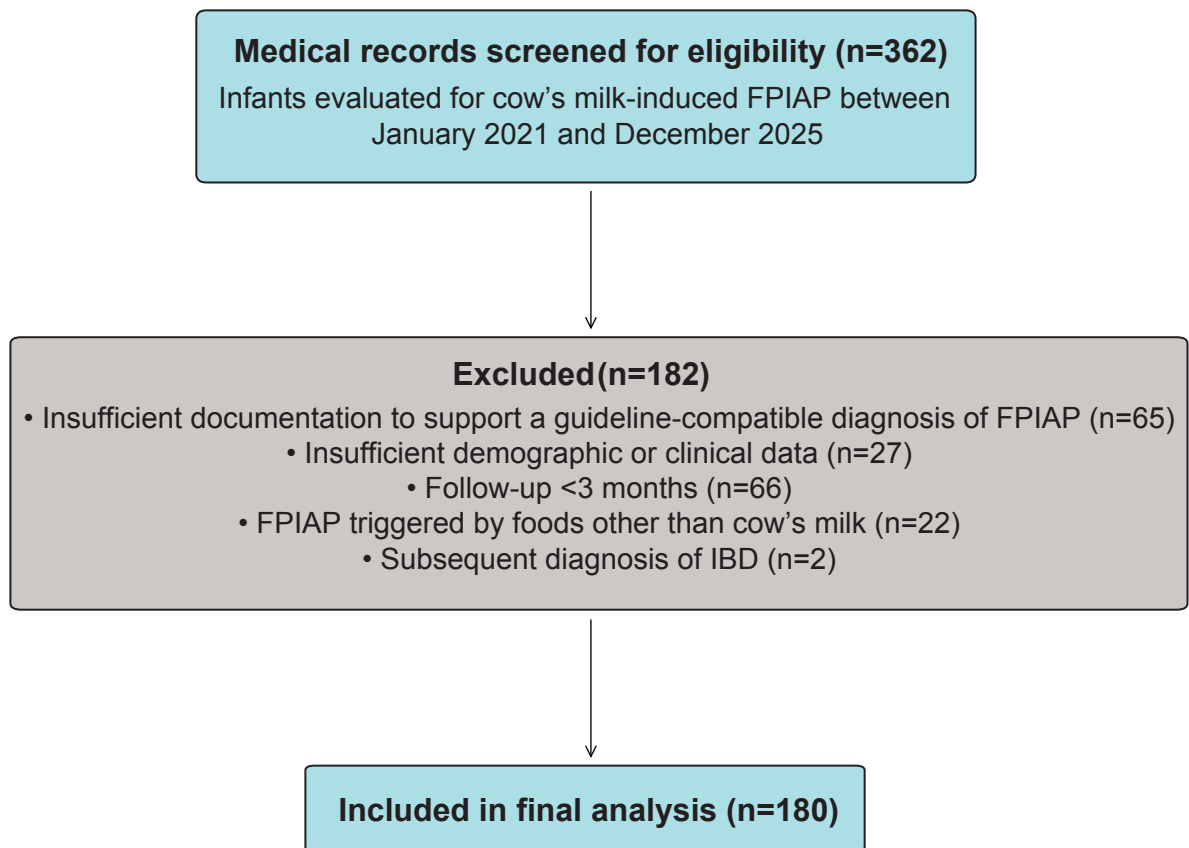

Supplement: Supplementary file 1 [file jcm-15-03862-s001.zip › figure s1 flowchart.pdf]
